# Utilization of gabapentin by people in treatment for substance use disorders in Belgium (2011–2014): a cross-sectional study

**DOI:** 10.1186/s13690-018-0254-8

**Published:** 2018-03-19

**Authors:** Luk Van Baelen, Karin De Ridder, Jérôme Antoine, Lies Gremeaux

**Affiliations:** Department of Public Health and Surveillance, Scientific Institute of Public Health, Brussels, Belgium, Rue Juliette Wytsmanstraat, 14, 1050 Brussels, Belgium

**Keywords:** Substance use disorders, Pharmacoepidemiological data, Health services, Belgium, Gabapentin

## Abstract

**Background:**

Although gabapentin has been licensed in the European Union only for neuropathic pain and epilepsy for patients who have partial seizures, it has also been prescribed in treatment for substance use disorders. Many studies report the potential risk of abuse of gabapentin by people with substance use disorders. The objective of this paper is to determine if people who have been in treatment for substance use disorders bought gabapentin in a time span that could indicate consumption at a dose that exceeded the maximum approved dose of 3600 mg/day.

**Methods:**

This analysis is the result of an observational cross-sectional descriptive study with matching. Two datasets were used and linked at individual level. Subjects were selected based on their first registration in the database of the Treatment Demand Indicator (TDI) between 2011 and 2014, without any exclusion criteria concerning nationality or age. Through linkage with the database of the InterMutualistic Agency (IMA) information on health service use and medication use was determined. In addition, each subject was matched on age, sex and place of residence to four comparators from the general population who were not in specialized treatment. The prevalence of gabapentin purchases in the period between 2008 and 2014 for both populations were compared. Quantification of the amount of gabapentin between two consecutive purchases was used as a proxy for potential abuse.

**Results:**

Out of 30,905 patients in treatment for substance use disorders 2.7% had bought at least once gabapentin in a public pharmacy or received it from a hospital pharmacy, compared to 0.7% in the comparison group (*n* = 122,142). In both populations, more than half of the patients bought only once or twice gabapentin and about 10.0% bought at least once gabapentin in a time span that could indicate potential abuse. A limitation of the study is that it is only based on reimbursed medication without clinical information.

**Conclusion:**

Through the linkage of the TDI-database and the database of the Belgian health insurance companies, no evidence was found for regular abuse of prescribed gabapentin in Belgium by people in treatment for substance use disorders.

## Background

In August 2006, the European Medicines Agency (EMA) granted a marketing authorization in all member states of the European Union for gabapentin. It was licensed for neuropathic pain and epilepsy for patients who have partial seizures [1]. Gabapentin is among the medications with the highest proportion of off-label use and has been prescribed for a range of conditions like bipolar disorder, peripheral neuropathy, diabetic neuropathy, complex regional pain syndrome, attention deficit disorder, restless legs syndrome, trigeminal neuralgia, periodic limb movement, sleeping disorders and migraine headaches [2]. In the majority of circumstances there is evidence that it is not the optimal treatment for these conditions [2], except for some very specific psychiatric disorders. Indeed, people with anxiety disorders might benefit from gabapentin [3], it has clear efficacy for alcohol dependence and relapse-related symptoms of insomnia, dysphoria and craving [3, 4], and it may be used in adjunctive treatment of opioid dependence [5, 6]. In a study by Bramness et al. [7] gabapentin was also successful in helping benzodiazepine users to reduce their consumption of benzodiazepine.

After the EMA granted marketing authorization, the pharmacological effect of gabapentin was quickly recognized by prescribers. For instance, between 2008 and 2012 gabapentin prescribing in the UK increased by 150%, to 3.5 million scripts [8]. Another report in the UK revealed that the prescription of gabapentin rose with 46% between 2011 and 2013 [9].

At the same time, reports mentioned the potential abuse of gabapentin by people with substance use disorders [9–14]. It was said to constitute a valid substitute for most common illicit drugs and this was a reason of concern [15–17].

The main objective of the study was to determine potential abuse by people in treatment for substance use disorders, i.e. whether they bought gabapentin in a time span that could indicate consumption at a dose that exceeded the maximum approved dose of 3600 mg/day. In addition, gabapentin purchases by people in treatment for substance use disorders were compared with purchases by users not in specialized treatment. Indeed, if people with substance use disorders were more susceptible to gabapentin abuse, it was expected that they would buy significantly more gabapentin within a time span that could indicate consumption at a dose that exceeded the maximum approved dose than users who were not in specialized treatment for substance use disorders.

## Methods

In this cross-sectional study data from two Belgian national health and population registers were used. Data from the Belgian Treatment Demand Indicator (TDI) database [18] were linked to pharmacoepidemiological and health service use data gathered through the seven Belgian health insurance agencies and consolidated in the InterMutualistic Agency database (IMA) [19–21], using the Belgian National Identification Number (NIN). This number is unique for every Belgian citizen and for other people living in Belgium with social security rights. 99% of the people living in Belgium have a NIN [19].

As described in detail by the research protocol [22], inclusion of subjects was defined by patients’ first treatment episode for substance use disorders between 2011 and 2014. An episode was defined as the period between the start of the treatment (i.e. the first face-to-face contact between a professional and the patient) and the end of activities in the context of the program prescribed. Subjects are patients who have sought treatment for substance use disorders within the reference period, without any exclusion criteria concerning nationality or age.

**Fig. 1:**
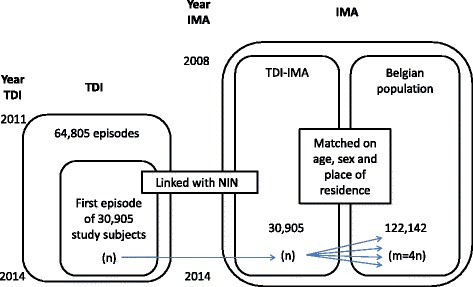
Set up of the linkage and matching procedure for subjects and comparators of the TDI-IMA database in Belgium

As illustrated by Fig. 1, between 1 January 2011 and 31 December 2014 64,805 episodes have been registered in TDI. However, patients could have had more than one treatment episode in the given reference period. In this case only data from the first registered episode were used in present analysis. Moreover episodes can be registered without NIN and this is the case for approximately 33% of data in TDI. Since the NIN is used to identify an individual, this means that the exact number of people who have been in treatment for substance use disorders between 2011 and 2014 remains unknown. All patients registered with a NIN who have been in treatment for substance use disorders between 2011 and 2014 have been confirmed eligible subjects (*n* = 31,638). Since 117 of them had had their first episode before 2011 and 616 could not be identified in the IMA-database, 30,905 subjects were included in the study.

In addition a group of peers has been selected from the general population who had not been in specialized alcohol or drug treatment between 2008 and 2014. Four comparators were matched on age, sex and place of residence to each subject in treatment for substance use disorders. Sex and age were considered to be basic matching variables. The potential confounding of place of residence is related to regional differences in health care regulation, health care seeking and access to specialized medical health care for substance use disorders as well as other differences that might be present, for instance caused by socio-economic status of the patients by region. Some of them were matched to more than one person who was in treatment for substance use disorders. As a result 122,142 individuals who were not in treatment for substance use disorders between 2008 and 2014 were matched to the 30,905 individuals in specialized treatment between 2011 and 2014.

Data was gathered on medication and health service use through the IMA database [20, 21] for the period between 1 January 2008 and 31 December 2014. Reimbursement of medication and use of health services is regulated in a very strict way in Belgium and purchases of medication such as gabapentin require a prescription by a physician in order to be delivered by public pharmacies.

Variables of interest and their source are given in Table 1. Details about all variables have been described in the research protocol [22]. All drugs in the IMA-database are classified according to the World Health Organization Anatomical Therapeutic Chemical Classification System (ATC) classification. Information on purchases of gabapentin (ATC-code N03AX12), as primary outcome, was obtained from the database, including the quantity in mg for every purchase. The daily dose for gabapentin was calculated per patient and per purchase by dividing the amount of the dispensed drug by the number of days in the interval between two consecutive dates on which the drug was bought in the period between 2008 and 2014. According to the label, for licensed therapeutic conditions the DDD for gabapentin is 1800 mg/day, which is the assumed average maintenance dose per day for gabapentin used for its main indication in adults and adolescents [23], whereas the maximum dose is 3600 mg/day, i.e. the equivalent of two DDDs. Although gabapentin has been prescribed up to 4800 mg/day in long-term clinical trials on refractory bipolar and unipolar mood disorders [24, 25], other studies on on-labeled and off-labeled use of gabapentin have used 3600 mg/day as the maximum dose. Since there is no therapeutic reason to presume that people in treatment for substance use disorders need a dose that exceeds the maximum dose for licensed conditions, the dose of 3600 mg/day has been maintained in the present study.

**Table 1:** Variables of interest taken from the Treatment Demand Indicator Database (2011–2014) and the InterMutualistic Agency Database (2008–2014)

Variables only available for subjects (Source: TDI)	
Treatment center	
Region where subject was treated	
Type of program	
Subject’s characteristics	
Nationality	
Educational attainment	
Professional situation	
Treatment episode for substance use disorders	
Date of inclusion (treatment starting date)	
Previous treatment episodes (yes/no)	
Source of referral	
Primary substance	
Patterns of use for primary substance	
Injecting status	
Variables available for subjects and comparators (Source: IMA)	
Patient characteristics	
Age	
Sex	
Place of residence	
Medication purchases	
Day, month and year of purchase of gabapentin	
Inpatient or outpatient delivery	
Product specificities (Defined Daily Doses (DDDs), Quantities Per Package (QPP), Quantities Per Unit (QPU))	

By quantifying the amount of gabapentin available between two consecutive purchases, it was possible to develop a proxy for potential abuse, defined as the use of gabapentin at a quantity exceeding the maximum dose. The cumulated number of times each patient exceeded this quantity resulted in the times of abuse per patient. Because consumption in inpatient services is strictly controlled, calculations of potential abuse of gabapentin was only limited to outpatient prescriptions. The same method was used for people in treatment for substance use disorders as for people who had not been in specialized treatment. The abovementioned matching procedure allowed comparing the results for people who were in treatment for substance use disorders with those of people who were not in specialized treatment.

Numbers and proportions were used to describe the characteristics for both populations. Using matched univariable and multivariable logistic regression models, associations were studied between sociodemographic variables, the filling of prescriptions that resulted in the patient being dispensed gabapentin, and being in specialized treatment or not. In the multivariable model all factors listed in the univariable model were included. Statistical analysis was performed using SAS software version 9.3 (SAS Institute Inc., Cary, NC). The reporting of this study conforms to the STROBE guidelines (see Appendix 1) [26].

## Results

As shown in Table 2, out of 30,905 patients who were in specialized alcohol and drug treatment between 2011 and 2014, 649 (2.7%) had been prescribed gabapentin at least once in the period between 2008 and 2014. Details about patients’ demographic characteristics are reported in Table 3 and Appendix 2. Almost two thirds of these patients reported an alcohol problem, 9.1% had problems with opioids and also 9.1% was in treatment for hypnotics and sedatives.

**Table 2:** Number of people who purchased gabapentin in Belgium between 2008 and 2014 who were in specialized alcohol and drug treatment or not

	In specialized treatment between 2011 and 2014		Not in specialized treatment between 2008 and 2014		
	N	%	N	%	OR (95%CI)
Prescribed gabapentin between 2008 and 2014	649	2.7%	872	0.7%	3.0 (2.7–3.3)
Not prescribed gabapentin between 2008 and 2014	30,256	97.3%	121,270	99.3%

**Table 3:** Profile of patients initiated on gabapentin in Belgium between 2008 and 2014 who were in specialized alcohol and drug treatment or not

	In specialized treatment (*N* = 649, 2.7%)		Not in specialized treatment (*N* = 872, 0.7%)	
	N	%	N	%
Sex				
Male	382	58.9%	503	57.7%
Female	267	41.1%	369	42.3%
Age categories				
15 y–19 y	5	0.8%	4	0.5%
20 y–29 y	48	7.4%	59	6.8%
30 y–39 y	135	20.8%	145	16.6%
40 y–49 y	184	28.4%	264	30.3%
50 y–59 y	188	29.0%	275	31.5%
≥ 60 y	89	13.7%	125	14.3%
Received prescription gabapentin				
only inpatient	88	13.6%	34	3.9%
only outpatient	337	51.9%	693	79.5%
in- and outpatient	224	34.5%	145	16.6%
Initiation to gabapentin				
in outpatient service	469	72.3%	789	90.5%
in inpatient service	180	27.7%	83	9.5%
Number of prescriptions received per patient –only outpatient prescriptions				
1	247	38.1%	411	47.1%
2	98	15.1%	137	15.7%
3	50	7.7%	43	4.9%
4	42	6.5%	48	5.5%
5	25	3.9%	23	2.6%
6–10	69	10.6%	87	10.0%
11–20	72	11.1%	62	7.1%
+20	46	7.1%	61	7.0%
Times of abuse (>3600mg/day) – only outpatient prescriptions				
0	505	90.0%	756	90.2%
1	36	6.4%	51	6.1%
2	10	1.8%	9	1.1%
3–10	6	1.1%	21	2.5%
11–20	3	0.5%	1	0.1%
+20	1	0.2%	0	0.0%
Total abusers	56	10.0%	82	9.8%
Total users (outpatient only)	561		838	

Out of 122,142 people who were not in specialized alcohol and drug treatment, 872 had been prescribed gabapentin at least once (0.7%) (Table 2). The only demographic characteristics available for people who were not in specialized treatment were age and sex, as reported in Table 3.

Patients in specialized treatment had an increased likelihood of having been filled a prescription for gabapentin compared to people who were not in alcohol and drug treatment (OR 3.0, 95% CI 2.7–3.3). For both groups, the median age category was 40 to 49 years and slightly more men than women were filled a prescription for gabapentin (Table 3).

**Fig. 2:**
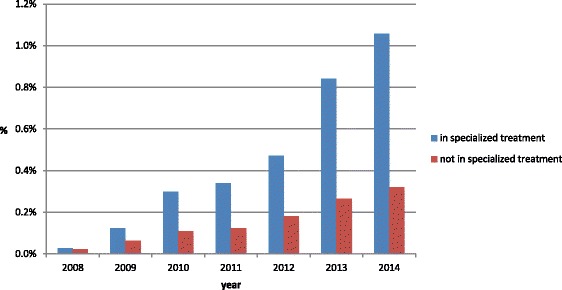
Proportion of patients who have been prescribed gabapentin per year on the total number of people in specialized treatment and not in specialized treatment

As shown in Fig. 2 the relative number of gabapentin users per year increased steadily from 0.03% in 2008 to 1.1% in 2014 among people in specialized treatment and during the same time span from 0.02% to 0.32% among those who had not been in specialized treatment.

Among gabapentin users, 72.3% of the patients in specialized treatment and 90.5% of people not in specialized treatment were initiated through outpatient services. Also later on, people who were not in alcohol or drug treatment had an increased likelihood of being dispensed gabapentin through outpatient services (Table 4). For both patients in specialized treatment and those not in specialized treatment, who were filled a prescription through outpatient services, more than half received only one or two prescriptions of gabapentin. Of those who were prescribed gabapentin through outpatient services, 56 (10.0%) of the people in specialized treatment and 82 (9.8%) of the people not in specialized treatment procured at least once gabapentin within a time span which could indicate the use of gabapentin at an estimated daily dosage that exceeded the maximum approved dose of 3600 mg/day. However, being in specialized treatment did not increase the likelihood of potential abuse of gabapentin (Table 4).

**Table 4:** Odds ratios and 95 % confidence intervals of being dispensed gabapentin in Belgium for people who were in specialized alcohol and drug treatment

	Univariable	Multivariable^a^
Received prescription gabapentin		
only inpatient	1.4 (0.9–2.3)	1.0 (0.5–1.8)
only outpatient	0.3 (0.2–0.4)	0.2 (0.1–0.3)
in- and outpatient (reference)	1	1
Initiation to gabapentin		
in outpatient service	0.3 (0.2–0.4)	0.9 (0.6–1.5)
in inpatient service (reference)	1	1
Number of prescriptions received per patient –only outpatient prescriptions		
1	0.7 (0.4–1.0)	1.5 (0.8–2.8)
2	0.9 (0.6–1.4)	1.5 (0.8–2.9)
3	1.5 (0.8–2.6)	2.7 (1.3–5.6)
4	1.0 (0.6–1.9)	1.6 (0.8–3.3)
5	1.2 (0.6–2.4)	1.5 (0.6–3.3)
6–10	1.0 (0.6–1.5)	0.9 (0.5–1.8)
11–20	1.4 (0.8–2.4)	1.6 (0.9–3.0)
+20 (Reference)	1	1
Times of abuse (>3600mg/day) – only outpatient prescriptions		
0 (Reference)	1	1
1	0.9 (0.5–1.3)	0.6 (0.4–1.0)
2	1.9 (0.6–4.6)	0.8 (0.3–2.6)
3–10	0.4 (0.2–1.0)	0.4 (0.1–1.0)
11–20	3.9 (0.4–38.1)	3.6 (0.3–44.0)
+20	–	–

## Discussion

Out of 30,905 patients in treatment for substance use disorders between 2011 and 2014, 2.7% had been prescribed gabapentin between 2008 and 2014. Compared to people who were not in specialized treatment in the same period, patients had an increased likelihood of having been filled a prescription for gabapentin. This is not unexpected, given the results of previous research: although gabapentin was licensed by the EMA as a drug for neuropathic pain and epilepsy for patients with partial seizures [1], it has also been reported to be efficient in alcohol dependence, abstinence and acute alcohol withdrawal [4], in the treatment and management of opiates [5] and to reduce the consumption of benzodiazepines [7]. When looking at outpatient support only, almost one in two people in specialized treatment received just one or two prescriptions for gabapentin. It could confirm off-label use of gabapentin in treatment of substance use disorders: gabapentin has been used in clinical trials on alcohol treatment [4, 27] and opioids [5] in protocols of less than one month, whereas protocols for neuropathic pain and epilepsy indicate long term treatment with titration schemes of two to three weeks to start up and a steady reduction in the consumption over at least one week at the end of the therapy [1]. This is also confirmed by the fact that more than 80% of the patients who were prescribed gabapentin were in treatment for alcohol, opioids or benzodiazepines as main substance.

However, if tackling withdrawal symptoms by off-label use of gabapentin is the main reason for the high number of people in specialized treatment with only one or two prescriptions of gabapentin, the question arises why people who were not in treatment for substance use disorders were more likely to get only one prescription. Indeed, 62.8% of them received only one or two prescriptions. One explanation might be that many people with alcohol or benzodiazepine dependence do not enter specialized treatment but prefer to seek help from their general practitioner: 90.5% of the people who were not in specialized treatment received the first prescription for gabapentin through outpatient services, which was significantly higher than for people who were in specialized treatment. Also later on, people who were not in treatment for substance use disorders were more likely to receive gabapentin only at outpatient health services.

The data of present study could not provide evidence to support the concern that people in treatment for substance use disorders are at risk for potential abuse of prescribed gabapentin, as suggested by other studies [9–17]. Indeed, slightly more than one in four of the people in treatment for substance use disorders who were prescribed gabapentin were filled more than five prescriptions and only 10.0% of the people in treatment for substance use disorders might have used gabapentin at a dose that exceeded the maximum approved dose of 3600 mg/day. More than 80% of them did so only once or twice. It can not be excluded that people who use gabapentin to reinforce or alter the effects of other drugs, buy gabapentin through online pharmacies, from other drug users or from their local drug dealer, as reported before [28, 29], but according to the data the phenomenon of medical and pharmaceutical shopping for gabapentin, whereby patients frequently go from doctor to doctor or from pharmacy to pharmacy, remains marginal in Belgium.

The main strength of the current research is the national coverage of the database and the availability of longitudinal data through the linkage of a database of people in treatment for substance use disorders with socio-economical, pharmacoepidemiological and health service data, as collected by the health insurance agencies.

Nonetheless, some limitations of the database have to be mentioned as well when interpreting the data.

First of all, there are some general limitations related to the linkage of the TDI- and IMA-database as discussed before in the research protocol [22]. Particularly the fact that the database does not contain information on patients’ diagnosis makes it difficult to distinguish between patients for whom gabapentin has been prescribed because of on-label conditions and others with off-label conditions such as substance use disorders. Indeed, people in TDI were in treatment for substance use disorders, but information on any coexisting on-label condition for which gabapentin can be prescribed such as neuropathic pain or epilepsy was missing. As such, it cannot be excluded that some of the patients in TDI with an alcohol or drug problem were prescribed gabapentin for on-label conditions and that any abuse of gabapentin was related to this specific condition rather than to the existing substance use disorders.

Secondly, the analysis was based on DDD, which is a theoretical construct, rather than a directly observed indicator such as Prescribed Daily Doses (PDD) or Consumed Daily Doses (CDD). Although these indicators reflect better actual consumption than DDD, in current study no information was available on the exact prescribed doses, on titration schemes that were used or on actual consumption rates. As such it might be that people have used gabapentin at a dose higher than prescribed by the physician, but since the cut-off of two times the DDD was used as a proxy, this kind of abuse remained unnoticed.

Finally, general practitioners did not participate in the TDI-registration and hence their work with people who seek treatment for substance use disorders is not reflected in the current data. Indeed, the data suggest that some people who were not registered in the TDI-database and for whom no codes of medication used for alcohol dependence (ATC N07BB) or opioid dependence (ATC N07BC) were recorded, were actually in treatment for alcohol or opioid dependence with a caregiver who did not participate in the TDI registration between 2011 and 2014. The IMA-database as such could not provide the necessary information since it is a register of data on reimbursed medication and services. Some medication specifically used in alcohol or opioid dependence is not reimbursed by the Belgian insurance system (e.g. naltrexone) or it is only reimbursed under strict conditions (e.g. nalmefene). Also benzodiazepines are not reimbursed. As such, it might be that some people who were not in specialized treatment were prescribed medication for alcohol, opioid or benzodiazepine dependence, but not correspondingly registered in the IMA-database. As a result any concomitant or prior prescription of gabapentin and non-reimbursed medication for alcohol dependence remained unidentifiable, meaning that some of the people who were not in specialized treatment actually were treated for alcohol dependence. As such it could be that gabapentin is used more frequently in treatment for substance use disorders than suggested by the data. This could have a slight influence on the number of patients with substance use disorders who might have used gabapentin at an estimated daily dosage that exceeded the maximum approved dose.

## Conclusion

The current study could not find any indication that people treated for substance use disorders used prescribed gabapentin frequently at a dose that exceeded the maximum approved dose of two times the DDD. These data reflect the regular procurements through pharmacies and hospitals and as such they do not exclude that people purchased gabapentin from local drug dealers or online pharmacies. However, in case of gabapentin the risk of medical shopping by people who were in treatment for substance use disorders in Belgium is considered to be minimal.

## Appendix 1

**Table 5:** STROBE Statement—Checklist of items Misuse of gabapentin by people in treatment for substance use disorders in Belgium: application of the TDI-IMA linkage [26]

	Item No	Recommendation	Where met and described, or if not met, reasons why not
Title and abstract	1	(*a*) Indicate the study’s design with a commonly used term in the title or the abstract	See abstract first sentence ‘Methods’ section.
		(*b*) Provide in the abstract an informative and balanced summary of what was done and what was found	See abstract ‘Methods’, ‘Results’ and ‘Conclusion’ section.
Introduction			
Background/rationale	2	Explain the scientific background and rationale for the investigation being reported	See paragraphs 1–3 from Background
Objectives	3	State specific objectives, including any prespecified hypotheses	See last paragraph Background
Methods			
Study design	4	Present key elements of study design early in the paper	See Methods paragraph 1
Setting	5	Describe the setting, locations, and relevant dates, including periods of recruitment, exposure, follow-up, and data collection	In Methods paragraph 1 and 2 the setting has been described as a Belgian national study based on data from TDI (1/1/2011–31/12/2014) and IMA (1/1/2008–31/12/2014).
Participants	6	(*a*) Give the eligibility criteria, and the sources and methods of selection of participants	Methods paragraph 2 and 3 (cases): ‘As described in detail… 30,905 subjects were included in the study’ and 4 (comparators): ‘In addition a group of peers… matched to the 30,905 individuals in specialized treatment between 2011 and 2014.’
Variables	7	Clearly define all outcomes, exposures, predictors, potential confounders, and effect modifiers. Give diagnostic criteria, if applicable	See Methods paragraph 6 and 7 for the definition of the outcome (use and misuse of gabapentin): ‘The daily dose of gabapentin was calculated… The same method was used for people in treatment for substance use disorders as for people who had not been in specialized treatment.’ Given the cross-sectional data, age and gender are potential confounders, as well as region, as described in Methods paragraph 4. To adjust for these confounders the cases were matched to a group of comparators. There is little information available on potential confounders or effect modifiers other than these. The exposure (problematic substance use) was available in TDI for people with substance use disorders. As mentioned in Methods, third paragraph, for comparators, exclusion criteria for matching (excluding for problematic substance use) are mentioned in: Van Baelen L, De Ridder K, Antoine J, Gremeaux L: Longitudinal pharmacoepidemiological and health services research for substance users in treatment: protocol of the Belgian TDI-IMA linkage. *Archives of Public Health*, in press.
Data sources/ measurement	8*	For each variable of interest, give sources of data and details of methods of assessment (measurement). Describe comparability of assessment methods if there is more than one group	As described in Methods, paragraph 6, variables of interest and their source are given in Table 1. Details about all variables have been described in: Van Baelen L, De Ridder K, Antoine J, Gremeaux L: Longitudinal pharmacoepidemiological and health services research for substance users in treatment: protocol of the Belgian TDI-IMA linkage. *Archives of Public Health*, in press.
Bias	9	Describe any efforts to address potential sources of bias	The potential confounding effect of age group, sex and region were taken into account when matching cases to comparators (Methods, fourth paragraph). Other sorts of bias are more difficult to address in this dataset, but we assess them in the Discussion. One source of bias could be the fact that 33% of patients with substance use disorders are registered without National Identification Number and its consequences for potential bias remain unknown (Methods, third paragraph).
Study size	10	Explain how the study size was arrived at	The study size (numbers of cases, numbers of comparators) are straightforwardly derived from the TDI-database and the IMA-database.
Quantitative variables	11	Explain how quantitative variables were handled in the analyses. If applicable, describe which groupings were chosen and why	The quantitative variables were used as available in both databases. No further manipulation was done, apart from the composite variable for use and misuse of gabapentin which is described in Methods paragraph 6: ‘The daily dose... The same method was used for people in treatment for substance use disorders as for people who had not been in specialized treatment.’
Statistical methods	12	(*a*) Describe all statistical methods, including those used to control for confounding	See Methods, last paragraph.
		(*b*) Describe any methods used to examine subgroups and interactions	Not applicable.
		(*c*) Explain how missing data were addressed	Data were only missing for descriptive variables. If data were missing for exposure or outcome variables (e.g. administrative errors), this information was not available.
		(*d*) If applicable, describe analytical methods taking account of sampling strategy	Not applicable.
		(*e*) Describe any sensitivity analyses	Not applicable.
Results			
Participants	13*	(a) Report numbers of individuals at each stage of study—eg numbers potentially eligible, examined for eligibility, confirmed eligible, included in the study, completing follow-up, and analysed	See Results, paragraph 1 and 2.
		(b) Give reasons for non-participation at each stage	Non-participation is not applicable, although some patients were registered without National Identification Number. This may cause bias, but no further information about potential direction is available.
		(c) Consider use of a flow diagram	Figure 1.
Descriptive data	14*	(a) Give characteristics of study participants (eg demographic, clinical, social) and information on exposures and potential confounders	Demographic characteristics are available in Table 3 and Appendix 1.
		(b) Indicate number of participants with missing data for each variable of interest	See Appendix 1.
Outcome data	15*	Report numbers of outcome events or summary measures	See Table 2 and Results paragraph 1 and 2.
Main results	16	(*a*) Give unadjusted estimates and, if applicable, confounder-adjusted estimates and their precision (eg, 95% confidence interval). Make clear which confounders were adjusted for and why they were included	See Table 3 and Results paragraph 3 and 4
		(*b*) Report category boundaries when continuous variables were categorized	See Table 3; the only categorized continuous variable was age, but categories were predefined. For age, we combined existing categories (e.g.20–24 and 25–29) into one separate category (e.g. 20–29). Because of low prevalence figures above 20 for ‘number of prescriptions’ and ‘times of misuse’, we made one category.
		(*c*) If relevant, consider translating estimates of relative risk into absolute risk for a meaningful time period	Not applicable.
Other analyses	17	Report other analyses done—eg analyses of subgroups and interactions, and sensitivity analyses	Not applicable.
Discussion			
Key results	18	Summarise key results with reference to study objectives	This is the main focus of Discussion, paragraph 1
Limitations	19	Discuss limitations of the study, taking into account sources of potential bias or imprecision. Discuss both direction and magnitude of any potential bias	This is the main focus of Discussion, paragraph 4–6
Interpretation	20	Give a cautious overall interpretation of results considering objectives, limitations, multiplicity of analyses, results from similar studies, and other relevant evidence	This is the main focus of Discussion, paragraph 1–3
Generalisability	21	Discuss the generalisability (external validity) of the study results	This is the main focus of Conclusion
Other information			
Funding	22	Give the source of funding and the role of the funders for the present study and, if applicable, for the original study on which the present article is based	This is the main focus of Funding

## Appendix 2

**Table 6:** Socio-demographic and substance use profile and use of other medication by patients in treatment for substance use disorders in Belgium, who have been prescribed gabapentin between 2008 and 2014, and a sub-sample of patients who might have used gabapentin at a dose that could indicate abuse

	Prescribed gabapentin (N = 649)		Abuse gabapentin (*N* = 56)	
	N	%	N	%
Region^a^				
Flanders	403	62.1%	36	64.3%
Wallonia	200	30.8%	16	28.6%
Brussels	46	7.1%	4	7.1%
Program type				
Medical Social Care Center	31	4.8%	3	5.4%
Specialized outpatient service	69	10.6%	4	7.1%
Crisis center	12	1.9%	0	0.0%
Therapeutic community	31	4.8%	2	3.6%
Mental health service	28	4.3%	2	3.6%
Psychiatric hospital	266	41.0%	24	42.9%
General hospital	212	32.7%	21	37.5%
Past treatment				
No	162	25.0%	12	21.4%
Yes	455	70.1%	42	75.0%
Unknown/missing	32	4.9%	2	3.6%
Source of referral				
Own initiative	293	45.2%	30	53.6%
Family or friends	97	15.0%	5	8.9%
Outpatient center for substance use disorders	10	1.5%	0	0.0%
General practitioner	99	15.3%	6	10.7%
Hospital or other medical service	93	14.3%	12	21.4%
Social service	5	0.8%	1	1.8%
Police or justice	36	5.6%	0	0.0%
Other	8	1.2%	2	3.6%
Unknown/missing	8	1.2%	0	0.0%
Education				
No	7	1.1%	0	0.0%
Primary education	108	16.6%	8	14.3%
Secondary education	383	59.0%	31	55.4%
Higher education	100	15.4%	11	19.6%
Unknown/missing	51	7.9%	6	10.7%
Main substance				
Opiates	5	0.8%	1	1.8%
Heroin	36	5.6%	0	0.0%
Methadone	9	1.4%	1	1.8%
Buprenorphine	1	0.2%	0	0.0%
Other opiates	8	1.2%	1	1.8%
Opioids (total)	(59)	(9.1%)	(3)	(5.4%)
Cocaine	27	4.2%	1	1.8%
Cocaine (other)	5	0.8%	0	0.0%
Cocaine (total)	(32)	(4.9%)	(1)	(1.8%)
Amphetamines	15	2.3%	0	0.0%
Stimulants (other)	2	0.3%	0	0.0%
Stimulants (total)	(17)	(2.6%)	(0)	(0.0%)
Hypnotics and sedatives	5	0.8%	0	0.0%
Barbiturates	2	0.3%	0	0.0%
Benzodiazepines	44	6.8%	4	7.1%
Other hypnotics and sedatives	8	1.2%	0	0.0%
Hypnotics and sedatives (total)	(59)	(9.1%)	(4)	(7.1%)
Cannabis	42	6.5%	2	3.6%
Alcohol	423	65.2%	45	80.4%
Other	17	2.6%	1	1.8%
Frequency of use main substance				
Not used in the last month	41	6.3%	1	1.8%
Once per week or less	30	4.6%	3	5.4%
Two to six times per week	90	13.9%	8	14.3%
Daily	451	69.5%	41	73.2%
Unknown/missing	37	5.7%	3	5.4%
Age first use main substance				
Median age	18		17.5	
Ever injecting behavior				
Ever injected but not currently	41	6.3%	2	3.6%
Currently injecting	18	2.8%	2	3.6%
Never injected	481	74.1%	40	71.4%
Unknown/missing	109	16.8%	12	21.4%
Year of start treatment				
2011	109	16.8%	9	16.1%
2012	174	26.8%	16	28.6%
2013	167	25.7%	12	21.4%
2014	199	30.7%	19	33.9%
Nationality				
Belgian	614	94.6%	53	94.6%
EU citizen but not Belgian	11	1.7%	3	5.4%
Non-EU citizen	10	1.5%	0	0.0%
Unknown/missing	14	2.2%	0	0.0%
Professional situation				
Regular job	83	12.8%	4	7.1%
Student	4	0.6%	1	1.8%
Economically non-active	426	65.6%	41	73.2%
Unemployed	85	13.1%	6	10.7%
Other	29	4.5%	4	7.1%
Unknown/missing	22	3.4%	0	0.0%

^a^Of all treatment programs participating in the TDI registration, 54% is located in Flanders, 32% in Wallonia and 14% in Brussels

## Abbreviations

ATC: Anatomical Therapeutic Chemical Classification System
DDD: Daily Defined Doses
EMA: European Medicines Agency
EMCDDA: European Monitoring Center for Drug and Drug Addiction
EU: European Union
IMA: Health Insurance Agency (InterMutualistisch Agentschap – Agence InterMutualiste)
NIN: National Identification Number
TDI: Treatment Demand Indicator
